# EPAC null mutation impairs learning and social interactions via aberrant regulation of miR-124 and Zif268 translation

**DOI:** 10.1186/1750-1326-7-S1-O12

**Published:** 2012-02-07

**Authors:** Ying Yang, Xiaogang Shu, Dan Liu, Lei Pei, Lingqiang Zhu, Qing Tian, Jianzhi Wang, Youming Lu

**Affiliations:** 1 Department of Pathophysiology, Key Laboratory of Neurological Disease of National Education Ministry and Hubei Province, Tongji Medical College, Huazhong University of Science and Technology, Wuhan, 430030, China

## Background

EPAC proteins are the guanine nucleotide exchange factors that act as the intracellular receptors for cyclic AMP. Two variants of EPAC genes including EPAC1 and EPAC2 are found to be expressed throughout the brain. But, their neurological functions have yet to be described.

## Method

We generate genetically-modified strains of mice with deficiency in expression of EPAC1 (EPAC1-/- mice), or EPAC2 (EPAC2-/- mice) or both EPAC1 and EPAC2 genes (EPAC-/- mice).

## Result and conclusion

We show that EPAC null mutation in the forebrain of mice impairs LTP of synaptic transmission that is paralleled with the abnormal spatial learning and social behaviors. This impairment is mediated in a direct manner by expressing miR-124, which binds to and inhibits Zif268 mRNA translation. Knockdown of miR-124 completely reverses the phenotypes observed in EPAC-/- mice, whereas over-expression of miR-124 mimics EPAC null mutation. Thus, miR-124 constitutes a critical epigenetic signaling downstream of EPAC proteins for regulation of learning and social interactions.

